# The Patients Assessment Chronic Illness Care (PACIC) questionnaire in The Netherlands: a validation study in rural general practice

**DOI:** 10.1186/1472-6963-8-182

**Published:** 2008-09-01

**Authors:** Michel Wensing, Jan van Lieshout, Hans Peter Jung, Jan Hermsen, Thomas Rosemann

**Affiliations:** 1 Scientific Institute for Quality in Healthcare, Radboud University Nijmegen Medical Centre, Nijmegen, The Netherlands; 2 General Practice Afferden, Afferden, The Netherlands; 3 Department of General Practice, University Hospital Zürich, Zürich, Switzerland

## Abstract

**Background:**

Many patients with chronic illness receive health care in primary care settings, so a challenge is to provide well-structured chronic care in these settings. Our aim was to develop and test a Dutch version of the PACIC questionnaire, a measure for patient reported structured chronic care.

**Methods:**

Observational study in 165 patients with diabetes or COPD from four general practices (72% response rate). Patients completed a written questionnaire, which included instruments for assessing chronic illness care (PACIC), evaluations of general practice (Europep), enablement (PEI), and individual characteristics.

**Results:**

The patients had a mean age of 68.0 years and 47% comprised of women. Twenty-two to 35% of responding patients did not provide answers to specific items in the PACIC. In 11 items the lowest answering category was used by 30% or more of the responders and in 6 items the highest answering category was used by this number of responders. Principal factor analysis identified the previously defined five domains reasonably well. Cronbach's alpha per domain varied from 0.71 to 0.83, and the intraclass coefficient from 0.66 to 0.91. Diabetes patients reported higher presence of structured chronic care for 14 out of the 20 PACIC items. The effect of patient evaluations of general practice on the PACIC score was positive (b = 0.72, p < 0.004), but the effect of patient enablement on the PACIC score was negative (b = -1.13, p < 0.000).

**Conclusion:**

A translated and validated Dutch version of the PACIC questionnaire is now available. Further research on its validity is recommended.

## Background

The Chronic Care Model is a conceptual framework that supports the evidence-based proactive and planned care for chronic diseases [1,2]. It has received widespread acceptance as a framework for improving the care of chronically ill patients. Measures of chronic care delivery are required to target efforts to improve chronic care and to monitor change of chronic care delivery over time. The Patient Assessment of Chronic Illness Care (PACIC) is a 20-item questionnaire for patients, which intends to measure chronic care delivery and which has been validated in USA for diabetes [3] and in Germany for osteoarthritis [4]. A version for chronic care in general practice in The Netherlands was not yet available, although many chronic patients receive most of their health care in general practice. Therefore the aim of our study was to develop a Dutch version of the PACIC instrument and test it on patients with diabetes or COPD in general practice.

## Methods

### Design and setting

An observational study was performed in randomly sampled patients from four general practices, which were situated in a rural area in the south-eastern part of The Netherlands. Two practices were single-handed and two practices were group practices. All practices were involved in a program to enhance structured diabetes care, while no such program existed for COPD care. Ethical approval was received for this study from the Arnhem-Nijmegen ethical committee.

### Study population

In each practice patients with diabetes mellitus and with COPD were sampled from the medical record system. An alphabetically ordered list of patient names was made, from which every second patients was included up to 30, except for one practice, which could provide only 20 COPD patients. A total of 230 patients was approached (120 diabetes patients and 110 COPD patients). Written questionnaires were sent by the practices, followed by postcard reminders three weeks later. Patients were invited to complete the questionnaire and return it anonymously in a prepaid envelope to the research institute. Informed consent was not explicitly asked, but implied when a patient returned the questionnaire.

### Measures

The questionnaire included the following measures. Patient assessment of chronic illness care (PACIC) was measured with a 20 item questionnaire, which used a five point response scale (ranging from 1 = 'almost never' to 5 = 'almost always') [3]. Higher scores mean more frequent presence of the aspect of structured chronic care. This instrument has five pre-defined domains: patient activation (3 items), delivery system/practice design (3 items), goal setting/tailoring (5 items), problem solving/contextual (4 items), follow-up/coordination (5 items). The English version was translated and culturally adapted in a structured procedure, including forward and backward translations, each by two independent researchers and then established in a consensus meeting with the four individuals involved. Next, face to face interviews were done with 15 elderly patients with chronic illness from one general practice. This led to substantial adaptations, mainly to simplify and clarify the questions, which were also discussed with the authors of the PACIC. Finally, we made two slightly different versions, one for patients with diabetes and one for patients with COPD (see Additional file 1). These versions different in two ways: the heading referred to diabetes or lung disease, and item 19 referred to lung physicians (for COPD patients) or internist, surgeon or ophmatologist (for diabetes patients). Aggregated mean scores for five domains and for the total instrument were calculated as described in previous research [3]. No scores per domain were determined for patients with missing values on on more than one third of the items in the domain.

Patient enablement (PEI) was measured with a six-item questionnaire (with three response categories: 0 = 'same or worse', 1 = 'better', 2 = 'much better') [5]. The range of the aggregated sum score was 0 to 12, with a higher score indicating a higher level of enablement. Respondents giving two or more missing values were excluded. Patient evaluations of general practice were measured with the Europep instrument, a 23-item internationally standardised and validated questionnaire (with a five point answering scale, ranging from 'poor' to 'excellent') [6]. For this study, we determined the overall mean value on the 17 dichotomized (excellent versus other values) items focused on clinical performance (Cronbach's alpha = 0.97). Respondents with more than 5 missing items were excluded. Finally the questionnaire contained questions on patient age, gender, highest education, and overall health status (single item with five point response scale, 'excellent to 'poor).

### Data-analysis

The analysis of the measurement properties of the PACIC was based on published quality criteria for questionnaires [7]. The content validity of the PACIC is based on the Chronic Care Model [1,2]. The interpretability of the instrument (the extent that qualitative meaning can be assigned to the qualitative scores) was based on the percentage of chronic patients who provided valid responses on each of the items. In addition, we checked for floor and ceiling effects in terms of percentage of patients using the most extreme (upper or lower) response categories.

Principal factor analysis (PCA, factors with eigenvalue > 1, varimax rotation) was applied to examine the number and type of domains in the instrument [8]. We determined the Kaiser-Meyer-Olkin Measure of sampling adequacy and the Bartlett's test of sphericity. Internal consistency (the extent to which items measure the same concept) was expressed in terms of Cronbach's alpha for each of five domains in PACIC and for the total PACIC instrument. Reliability was expressed as an intra class coefficient (ICC, absolute agreement), which was based on variation between patients divided by total variation (taking patients random and items fixed). Values > 0.70 for alpha and ICC were considered acceptable [7].

The analysis of construct validity was based on the following hypotheses. We expected that higher PACIC scores, reflecting patient perceived presence of structured chronic care, would be positively related to both patients' perceived enablement after the latest visit to the GP and to patients' overall evaluations of general practice. To verify this expectation, we used linear regression analysis [9] with PACIC scores as dependent factor, enablement or evaluation as independent factor, and patient age and gender also included in the model. All data-analysis was done with SPSS 14.

## Results

In total, we received completed questionnaires from 165 patients: 88 diabetes patients (response rate 73%) and 77 COPD patients (70%). Table 1 provides descriptive information on the patient samples. The patients' mean age was 68 years); only a minority had medium or high education (36%); and just over half of them (55%) reported a good or excellent health status. Diabetes patients were, compared to COPD patients, more frequently female (57 versus 35%). More diabetes patients than COPD patients (66 versus 41%) reported good to excellent health status.

**Table 1:** Patient characteristics (n = 165)

	Total population	Diabetes patients (n = 88)	COPD patients (n = 77)
Mean age (SD)	68.0 (10.3)	68.8 (8.9)	67.2 (11.7)
Percentage women	47%	57% *	35%
Percentage with medium or high education	36%	34%	38%
Percentage with good to excellent health status	55%	66% *	41%
Mean sum score on patient enablement (PEI)	8.7 (2.9)	8.4 (3.0)	9.1 (2.7)
Percentage of patients who evaluated clinical performance as 'excellent' (Europep)	57%	60%	54%

* P < 0.05 of difference between diabetes patients and COPD patients.

Table 2 provides descriptive information on the PACIC items. Not all responders had completed all items of the PACIC questionnaire. The percentage of non-responders of all patients varied between 22 and 35%. Three items (numbers 15, 17 and 20) had 30% or more non-responders. The percentage of responders who used the lowest answering category (indicating complete absence of structured chronic care) varied from 7 to 76%, and was in 11 items 30% or higher. The percentages of responders who used the highest answering category (indicated complete presence of the aspect) varied from 10 to 54%, and was in 6 items 30% or higher.

**Table 2:** Descriptive information on PACIC items

	Item non-response in total study population (n = 165)	Floor and ceiling effects in total study population (n = 165)		% of responders reporting mostly/always present	
		% of responders in lowest response category (absence)	% of responders in highest response category (presence)	Diabetes patients (n = 88)	COPD patients (n = 77)

**Patient activation**					
1 Asked about my ideas when we made a treatment plan	24	21	21	59	40
2 Given choices about treatment to think about	26	25	20	54	40
3 Asked to talk about any problems with my medicines or their effects	24	20	28	54	52
**Delivery system/practice design**					
4 Given a written list of things I should do to improve my health	23	39	24	54	15
5 Satisfied that my care was well organized	23	7	54	88	75
6 Shown how what I did to talke care of my illness influenced my condition	25	18	46	72	50
**Goal setting/tailoring**					
7 Asked to talk about my goals in caring for my illness	26	30	16	48	25
8 Helped to set specific goals to improve my eating or exercise	22	19	35	73	27
9 Given a copy of my treatment plan	26	61	21	36	10 *
10 Encouraged to go to a specific group or class to help me cope with my chronic illness	27	76	10	21	2
11 Asked questions, either directly or on a survey, about my health habits	27	53	18	40	12
**Problem solving/contextual**					
12 Sure that my doctor or nurse thought about my values and my traditions when they recommended treatments to me	27	29	32	63	51
13 Help to make a treatment plan that I could do in my daily life	27	40	29	60	29
14 Helped to plan ahead so I could take care of my illness even in hard times	28	38	26	58	31
15 Asked how my chronic illness affects my life	30	38	23	43	29
**Follow-up/coordination**					
16 Contacted after a visit to see how things were going	26	52	16	32	19
17 Encouraged to attend programs in the community that could help me	30	78	10	19	4
18 Referred to a dietician, health educator, or counselor	26	40	45	75	13
19 Told how my visits with other types of doctors, like consultant or surgeon, helped my treatment	23	28	43	68	41
20 Asked how my visits with other doctors were going	35	29	20	46	27

The factor analysis identified five factors (explaining 70% of the variation; KMO = 0.844; Bartlett's test of spherity p = 0.000), which mostly confirmed the internal consistency for three of the five pre-defined domains (Table 3). The items for the remaining two domains, delivery system/practice design and follow-up/coordination, were scattered across domains.

**Table 3:** Factor loadings in rotated factor solution

**Items**	1	2	3	4	5
1 Asked about ideas	0.159	0.697	0.255	0.324	0.072
2 Given choices	0.145	0.767	0.150	0.116	-0.005
3 Talk about problems	0.170	0.770	0.221	-0.051	0.188
4 Given written list	-0.043	0.284	0.475	0.553	0.250
5 Care well organized	0.140	0.206	0.783	-0.022	0.033
6 Influence my condition	0.368	0.283	0.698	0.149	0.046
7 Talk about goals	0.364	0.442	0.305	0.379	-0.076
8 Set goals	0.425	-0.002	0.526	0.443	0.240
9 Given treatment plan	0.277	0.246	0.106	0.675	-0.097
10 Go to group or class	0.341	0.033	-0.047	0.133	0.798
11 Questions health habits	0.637	0.103	0.065	0.360	0.043
12 Values and traditions	0.706	0.255	0.099	0.021	0.054
13 Could do in daily life	0.774	0.122	0.326	0.194	0.072
14 Helped to plan ahead	0.642	0.322	0.292	0.074	0.219
15 How illness affects life	0.385	0.593	0.033	0.197	0.166
16 Contact after visit	0.455	0.173	0.224	0.110	0.187
17 Attend programmes	0.024	0.219	0.227	0.105	0.812
18 Referred	0.225	0.010	-0.045	0.702	0.293
19 Other doctors	0.684	0.166	0.020	0.192	0.150
20 Asked about visits	0.388	0.487	0.003	-0.001	0.380

Despite this partial support for the pre-defined factor structure in the PACIC instrument. Cronbach's alpha's and ICCs were above our threshold of 0.70 for the overall measure and for most pre-defined domains (Table 4). Lower than threshold values were identified for the ICCs in the domains delivery system/decision support and follow-up/coordination. The association of the aggregated Europep score and PACIC domains and overall score were all positive, as expected. However, higher enablement in patients was associated with lower scores on PACIC domains and overall score, as opposed to our expectation.

**Table 4:** Information on the PACIC domains and overall PACIC score

	Overall PACIC score	Patient activation	Delivery system/practice design	Goal setting/ tailoring	Problem solving/ contextual	Follow-up/ coordination
Number of items in the domain	20	3	3	5	4	5
Available sample for data-analysis	114	130	132	123	118	124
Patients with missing scores on more than one third of the items (% of total number of patients)	31%	21%	20%	25%	28%	25%
Mean value (SD) for all responders	2.9 (1.0)	3.2 (1.3)	3.5 (1.2)	2.5 (1.1)	3.3 (1.4)	3.1 (1.1)
Mean value (SD) for diabetes patients	3.2 (1.0)	3.4 (1.2)	3.9 (1.1)	2.9 (1.1)	2.5 (1.3)	2.0 (0.8)
Mean value (SD) for COPD patients	2.3 (0.8)	3.0 (1.3)	3.0 (1.2)	1.8 (0.8)	3.0 (1.4)	2.7 (1/1)
Effect of aggregated Europep score (b coefficient, p-value)	0.72 (p < 0.004)	0.88 (p < 0.003)	0.88 (p < 0.002)	0.50 (p < 0.064)	0.87 (p < 0.011)	0.74 (p < 0.009)
Effect of aggregated enablement score (b coefficient, SD)	-1.13 (p < 0.000)	-0.06 (p < 0.801)	-0.15 (p < 0.000)	-1.13 (p < 0.001)	-0.20 (p < 0.000)	-0.08 (p < 0.030)
Cronbach's alpha for internal consistency	0.93	0.85	0.75	0.81	0.87	0.71
Intra class coefficient (absolute agreement)	0.91	0.85	0.66	0.76	0.86	0.66

## Discussion

This study showed that the Dutch version of the PACIC instrument had mixed measurement properties when applied for assessing diabetes care and COPD care in general practice in a rural setting. The five previously defined domains were confirmed and their internal consistency was good. The correlation with patient evaluations of general practice was positive, and diabetes patients reported higher presence of structured chronic care than COPD patients as expected. However, substantial numbers of patients did not provide answers to the PACIC questions, although they returned the questionnaires and completed other parts of the questionnaire reasonably well. Also, we found that a number of items might have floor or ceiling effects. A surprising finding was that better scores for chronic care were linked to lower patient reported enablement after the latest consultation in general practice.

The mean scores on the PACIC domains and total instrument were similar to those found in diabetes patients in the USA [3], but higher than those found in patients with osteoarthritis in Germany [4]. The PACIC scores for diabetes patients in The Netherlands may be explained by the attention for enhancing structured diabetes care in recent years. For instance, there is no such attention for osteoarthritis, so we would expect similar scores for this condition compared to scores found in Germany. Despite the differences, our findings regarding measurement properties were similar to those found in Germany [4].

Obviously, the study had a number of limitations. The patient sample was relatively small, and only four general practices from a rural setting were involved, but it was not our aim to generalize the descriptive figures. It is difficult to speculate on how the validation results could be affected by the rural setting. Criterion validity, test-retest reproducibility and responsiveness to change could not be analysed. A substantial proportion of the patients used the lowest answering category, which may indicate a floor effect of the measure (inability to discriminate between patients). We suggest, however, that the scores might perfectly reflect reality – a complete absence of specific aspects of structured chronic care. The high number of non-responders was worrying. An explanation for the non-response may be a perceived lack of relevance of the aspects covered by the items. Some of the aspects covered in the PACIC instrument may be unknown or not relevant to many chronic patients in general practice in The Netherlands. A second explanation may be that the non-response actually implies absence of the aspects mentioned in the PACIC questionnaire – but we think we cannot be certain about such inferences. A final explanation for this is translation problems. A direct translation of the English questions into the Dutch language did not result in understandable language, so we had to rephrase the items quite substantially. Despite this, the final questionnaire might have remained too difficult for many patients.

We can only speculate about the (weak, but 5 out of 6 times highly significant) negative association between patient enablement with the latest visit in the practice and PACIC scores. Perhaps patients with a stronger internal health locus of control and better self-management 'ask' less for enablement, so that they do not need help in general practice regarding the aspects covered by PACIC. Enablement and structured chronic care (including patient activation) may be fundamentally different concepts, as opposed to our expectations beforehand. The finding might also suggest that structured chronic care could have some negative consequences, despite its intention to enhance self-management in patients.

## Conclusion

A validated Dutch version of the PACIC instrument is now available. Further research into its validity is recommended, particularly with respect to the high number of non-responders and the counterintuitive finding regarding patient enablement. Also, the questionnaire needs to be tested in other settings than primary care, before using it in those settings.

## Competing interests

The authors declare that they have no competing interests.

## Authors' contributions

MW initiated, designed and coordinated the study, carried out data-analysis and wrote the manuscript. JvL contributed to the development of the Dutch version of PACIC and checked the tables in the manuscript. HPJ and JH organized and carried out the data-collection. All authors read earlier versions of the manuscript, provided critical comments, and approved the final manuscript.

## Pre-publication history

The pre-publication history for this paper can be accessed here:



## Supplementary Material

Additional file 1 Dutch version of the PACIC. The PACIC questionnaire in Dutch language, which we have used in our study. Click here for file
