# Supplementary material for: The Patients Assessment Chronic Illness Care (PACIC) questionnaire in The Netherlands: a validation study in rural general practice
Source: BMC Health Serv Res. 2008 Sep 1;8:182. doi: 10.1186/1472-6963-8-182 (PMC2538520; doi:10.1186/1472-6963-8-182)
Supplement: Additional file 1 — Dutch version of the PACIC. The PACIC questionnaire in Dutch language, which we have used in our study. [file 1472-6963-8-182-S1.doc]

**Dutch version of the PACIC**

Als ik kijk naar de zorg voor mijn longziekte /diabetes die ik de afgelopen 6 maanden ontvangen heb vanuit de huisartspraktijk, kan het het volgende zeggen:

1. Mijn behandelaar (huisarts, verpleegkundige of praktijkassistente) heeft mij gevraagd naar mijn ideeën toen we een behandelplan maakten.
2. Ik kreeg keuze’s over de behandeling om over na te denken.
3. Mijn behandelaar (huisarts, verpleegkundige of praktijkassistente) heeft mij gevraagd om te praten over eventuele problemen met mijn medicijnen of hun werking.
4. Ik kreeg een schriftelijke lijst van dingen die ik zou moeten doen om mijn gezondheid te verbeteren.
5. Ik was tevreden over de organisatie van mijn zorg.
6. Mijn behandelaar heeft mij uitgelegd hoe de aandacht die ik besteed aan mijn ziekte van invloed is op mijn toestand.
7. Mijn behandelaar heeft me gevraagd om te praten over mijn doelen in de zorg voor mijn ziekte.
8. Mij behandelaar (huisarts, verpleegkundige of praktijkassistente) heeft me geholpen op specifieke doelen te stellen om mijn eetgedrag of lichaamsbeweging te verbeteren.
9. Ik heb een kopie van mijn behandelplan gekregen.
10. Mijn behandelaar heeft me aangemoedigd om naar een groep of cursus te gaan speciaal om mij te helpen om te gaan met mijn chronische ziekte.
11. Ik heb vragen gekregen, persoonlijk of via een vragenlijst, over mijn leefgewoonte’s.
12. Ik was er zeker van dat mijn arts of verpleegkundige rekening gehouden heeft met mijn waarden en mijn tradities toen deze mij een behandeling adviseerde.
13. Ik ben geholpen met het maken van een behandelplan dat ik kan uitvoeren in mijn dagelijks leven.
14. Mijn behandelaar heeft met geholpen om plannen te maken zodat ik kan omgaan met mijn ziekte, zelfs in moeilijke tijden.
15. Mijn behandelaar heeft me gevraagd hoe mijn chronische ziekte mijn leven beïnvloedt.
16. Na een praktijkbezoek werd er contact met me opgenomen om te zien hoe het met me ging.
17. Ik ben aangemoedigd om programma’s in de wijk te bezoeken die mij zouden kunnen helpen.
18. Ik ben verwezen naar een diëtist, gezondheidsvoorlichter of maatschappelijk werker.
19. Mijn behandelaar heeft me uitgelegd hoe contacten met andere artsen, zoals de longarts (een chirurg oogarts of internist), mijn behandeling ten goede zou kunnen komen.
20. Mijn behandelaar heeft me gevraagd hoe mijn bezoeken aan andere artsen gegaan zijn.
